# Bioprofiling TS/A Murine Mammary Cancer for a Functional Precision Experimental Model

**DOI:** 10.3390/cancers11121889

**Published:** 2019-11-27

**Authors:** Carla De Giovanni, Giordano Nicoletti, Lorena Landuzzi, Arianna Palladini, Pier-Luigi Lollini, Patrizia Nanni

**Affiliations:** 1 Department of Experimental, Diagnostic and Specialty Medicine, Alma Mater Studiorum University of Bologna, I-40126 Bologna, Italy; carla.degiovanni@unibo.it (C.D.G.); arianna.palladini@unibo.it (A.P.); patrizia.nanni@unibo.it (P.N.); 2 Laboratory of Experimental Oncology, IRCCS Istituto Ortopedico Rizzoli, I-40136 Bologna, Italy; giordano.nicoletti@fastwebnet.it (G.N.); lorena.landuzzi@ior.it (L.L.)

**Keywords:** TS/A, murine mammary cancer, preclinical models, gene therapy, metastases, immunotherapy

## Abstract

The TS/A cell line was established in 1983 from a spontaneous mammary tumor arisen in an inbred BALB/c female mouse. Its features (heterogeneity, low immunogenicity and metastatic ability) rendered the TS/A cell line suitable as a preclinical model for studies on tumor–host interactions and for gene therapy approaches. The integrated biological profile of TS/A resulting from the review of the literature could be a path towards the description of a precision experimental model of mammary cancer.

## 1. Introduction

Precision medicine in clinics is an evolving concept which goes beyond mere genomic medicine and means matching individual patients with medicine [1]. According to these premises, in an experimental environment a precision cancer model should mean matching the appropriate preclinical model with target biology study [2]. Preclinical models of mammary cancer of increasing complexity have been proposed, including transplantable murine tumors, gene-driven mammary carcinogenic models, human cell lines grown in vitro or in vivo as xenografts and patient-derived xenografts and organoids (see Section 6 for a comparative discussion) [3,4]. Each model remains an approximation [2], with advantages and disadvantages depending on the specific aim of the study. The main advantage of transplantable murine mammary tumors consists of allowing mechanistic studies on tumor–host interactions, like those focusing on the role of microenvironment, the metastatic process and the immune response. A deep knowledge of a preclinical model, where literature studies are collected and retrospectively examined as a whole, like an individual patient’s medical record, can help in a better design of experimental approaches. The aim of this review is the biological profiling of a popular model of murine mammary cancer (TS/A) for a better understanding and modeling of a complex pathology like human breast cancer [5].

## 2. The Dawn of Murine Models for Tumor–Host Interactions

At the beginning of the 1980s, metastases and tumor–host interaction studies mostly took advantage of a few tumor cell lines, established and subcultured for many years, such as 3LL Lewis lung carcinoma and B16 murine spontaneous melanoma [6]. Through the intravenous injection of B16 cells, metastatic deposits to lungs and other organs could be easily obtained, allowing for important advancements in understanding post-intravasation late phases of the metastatic process. However, the B16 parental cell line was almost incapable of disseminating from a locally-growing tumor, and therefore it did not adequately model the invasion and intravasation phases. Moreover, the non-epithelial origin of B16 melanoma impeded inferences about the behavior of epithelial tumors. At the same time, some rodent cell lines were already being used as models for mammary cancer, but most of them were either carcinogen- or virus-induced [7,8]. These models did not undergo a long natural history in the host, in which they arose, and generally had a high immunogenicity due to the expression of strong tumor-associated antigens. Likely due to these features, they generally gave too optimistic results when used to study antitumor immune responses or immunotherapeutic approaches [9].

In this landscape, in 1983 we described a new cell line, TS/A, derived from a mammary tumor spontaneously arisen in a 20 month-old BALB/c inbred mouse strain [10]. The TS/A cell line exhibited some features typical of human breast cancer, which prompted its use as a preclinical model, such as the low immunogenicity, the ability of local tumors to give rise to distant metastases and the heterogeneity, well evident both of morphology and metastatic ability. The TS/A cell line (also referred to as TSA or TS/A-pc, see [11,12] and below) and its clones were distributed to many laboratories worldwide and were employed for different applications, such as studies on malignant phenotype, pharmacologic therapeutic approaches, antitumor immune response and as a gene therapy model.

A list of research studies exploiting the TS/A cell line or its cell variants is reported in the Supplementary Table S1. It includes (up to 2018) 276 research papers where TS/A was used as model system and 19 papers where it was a control model. Reviews reporting results obtained with TS/A and citations of the TS/A paper are also listed in the Supplementary Table S1.

This review aims at profiling the main biological features of the TS/A model system resulting from literature research papers (Table 1). The two research areas where TS/A-based models yielded important results will be discussed in depth: tumor–host interactions and experimental gene therapy.

## 3. Bioprofiling TS/A Cell Line

**Figure 1:**
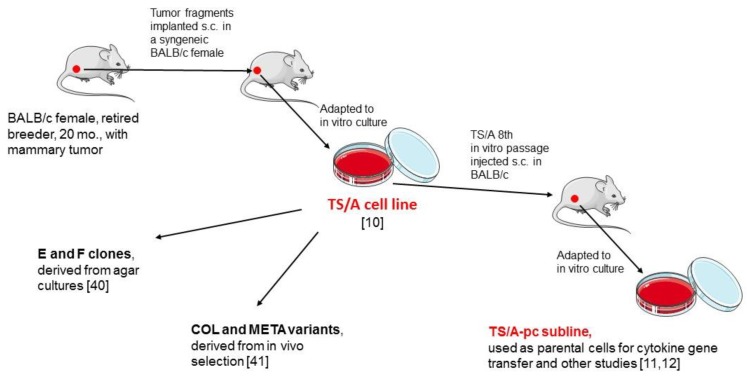
Origin of the TS/A cell line and variants. For pictures see [51].

The mammary tumor originating the TS/A cell line was isolated in a 20 months-old BALB/c female retired breeder and was described as a moderately differentiated adenocarcinoma [10]. Its first in vivo passage into a healthy BALB/c female was adapted to in vitro culture and named TS/A (Figure 1). Several clones and cell variants were derived from TS/A and distributed worldwide. In particular, a TS/A subline was chosen by Guido Forni (University of Turin) for a large collaborative endeavor as the recipient cell for the systematic transduction of a large series of genes coding for immune modulators; such subline was referred to as TS/A-pc (from “parental cells”). TS/A and TS/A-pc share most features reviewed here, and some kind of drift occurred during the extensive amplification of TS/A-pc. Throughout this review, we will refer to the TS/A model system on the whole, and therefore incorrect terminology (such as TSA, TS/a, and so on) has been systematically corrected to “TS/A”. However, the Supplementary Table reports exactly the TS/A cell variant used in each referenced paper.

The tumor from which the TS/A cell line was derived likely had a long natural history in its host of origin. When tested in a growth-excision test, TS/A cells did not confer protection against a second challenge, thus showing a low immunogenicity [10], thereafter confirmed in other studies (see, for example, [23]). Such a low immunogenicity was the basis for a huge number of immunopotentiation studies, most of which exploiting gene therapy approaches (see next section).

TS/A cells express the gp70env product of an endogenous retrovirus whose AH1 immunodominant class I epitope could be recognized by cytotoxic T lymphocytes through presentation by H-2L^d^ [25]. Gp70env antigen is shared by other murine cell lines, such as the colorectal cancer cell line CT26. The down-regulation of the L^d^ observed in TS/A cells [52] is likely due to the immunoediting process leading to evasion from the host immune response.

TS/A exerts a suppressive effect on the host immune response through several mechanisms, such as a selective loss of STAT5a/b expression in T and B lymphocytes [26], the production of transforming growth factor β1 (TGF-β1) [18], the induction of regulatory T cells [27], natural killer resistance [36] and the production of colony stimulating factors (CSFs) that deeply subvert hematopoiesis [14,15], giving rise to splenomegaly, leucocytosis and to tumor-infiltrating myeloid-derived suppressor cells (MDSC) [29,33,53].

When injected subcutaneously into syngeneic BALB/c mice, TS/A cells gave rise to local tumors rapidly disseminating to the lungs. Metastases could also be obtained after injection of TS/A cells by the intravenous route, thus allowing a comparison between the dynamics of the early and late phases of the metastatic process [10,41]. Metastases to lungs and liver have also been obtained by orthotopic cell injection [54]. Like other mammary carcinoma cell lines, the growth of micrometastases at distant organs was found to involve the formation of filopodium-like protrusions mediated by FAK/ERK and Rif/mDia2 signaling [54].

**Figure 2:**
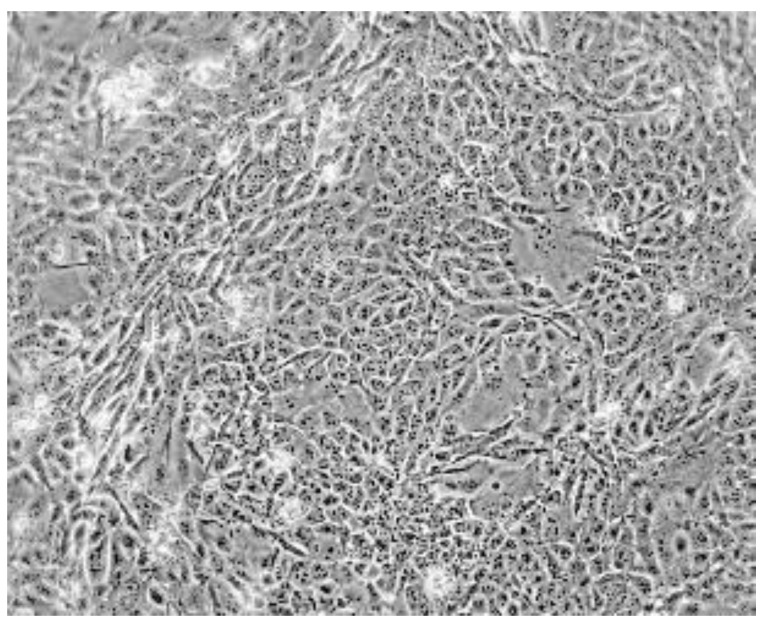
Morphology of the TS/A cell line in adherent culture (phase contrast, ×100).

Heterogeneity of TS/A cells was observed in adherent cultures, with areas of epithelial-like and fibroblast-like morphology (Figure 2), and in anchorage-independent cultures [10,40]. Subcloning from agar cultures allowed the isolation of two types of cell clones, both tumorigenic and metastatic, but with markedly different metastatic power [40]. Unexpectedly high-metastatic clones had a prevalent epithelial morphology, compared to the fibroblast-like pattern of low-metastatic clones. Gene expression profiling of several murine mammary cancer cell lines showed clustering of TS/A-E1 (a high-metastatic clone) with high-claudin expressors [13]. Our data on gene expression profiling of TS/A clones showed that claudin-3 was the top overexpressed gene in high-metastatic clones (about 90-fold expression over low-metastatic clones), while low-metastatic clones overexpressed nme4 and necdin, two putative metastasis suppressor genes (our unpublished results). These data suggest that metastatic ability is not always a consequence of epithelial–mesenchymal transition but can also be acquired in an epithelial-like differentiation context.

The TS/A cell line has a triploid karyotype [20] and carries a mutated p53 at codon 270 [21]. About a third of the cells express the cancer stem cell marker Sca-1 [43]. In our laboratory, the expression of Sca-1 (also known as Ly6A) was almost negative, but inducible by IFN-γ [12]. TS/A cells express estrogen receptor [10] and endogenous murine p185-erbB2 product [39]. Its use as a negative HER2/neu mammary cancer cell line in studies on HER2/neu transgenic models relies upon the negativity to the reagent specifically recognizing rat HER2/neu.

## 4. Tumor–Host Interaction Studies

TS/A-induced tumors have a rich and heterogeneous infiltrate comprising granulocyte and monocyte/macrophage subpopulations, whose relative proportions change during tumor progression [55], in agreement with the known plasticity of myeloid cells. Several subpopulations contribute to maintainance of a tumor-promoting microenvironment in TS/A, as well as in many other murine and human tumors [56], with a variety of mechanisms. Alternatively-activated M2 macrophages are strong producer of the immunosuppressive cytokine IL10 and of several chemokines recruiting Treg, Th2, eosinophils and basophils [56]. MDSC are heterogeneous immature CD11b+/Gr-1+ populations [57], with immunosuppressive function. Both M2-polarized macrophages and MDSC have been investigated in the TS/A model system, along with strategies to circumvent tumor promotion, pushing infiltrate cells towards more differentiated, activated cells.

In the TS/A model, the induction of M2 tumor-associated macrophages was mediated by the expression of the CD20 homolog *MS4A8A* gene [58]. In TS/A tumors, M2-polarized macrophages were more abundant and more proangiogenic in hypoxic tumor areas [55]. The M2 immune suppressing phenotype was switched to an anti-tumor M1 phenotype through the in vivo adenoviral gene transfer of the chemokine CCL16 [59]. Alternatively-activated M2 macrophages expressed highly restricted, individual-specific, combinatorial T cell receptor-αβ immunoreceptors, suggestive of an adaptive response of macrophages to the tumor [60]. In TS/A, as well as in a variety of other murine and human tumors, alternatively-activated M2 tumor-associated macrophages expressed a multifunctional scavenger receptor named stabilin-1 involved in endocytic and phagocytic clearance of “unwanted-self” components, including soluble component of extracellular matrix SPARC (a tumor-inhibiting agent). Stabilin-1 was found to play a tumor promoting role in the TS/A model likely through enhanced clearance of SPARC [61].

A major component of TS/A infiltrate consisted of MDSC [53], which correlated with the production of CSFs by TS/A cells [14,29]. Immature myeloid progenitors can be released in the bloodstream, giving rise to peripheral leukocytosis and splenomegaly [14,15]. MDSC suppressed antigen-activated T lymphocytes through apoptosis induction [28,29], and suppressed NK cytotoxicity [62], with mechanisms involving nitric oxide [30]. Impaired anti-tumor immune response in aging can take advantage of an increased MDSC infiltrate [32]. MDSC expressed Fas–FasL and caspases, suggesting that Fas–FasL apoptosis regulated MDSC survival [33,34] and proposing new potential therapeutic options. MDSCs are key drivers of resistance to antiangiogenic therapy, but all-trans retinoic acid was able to induce differentiation of MDSC into mature cells, thus increasing the efficacy of the antiangiogenic therapy [63].

In the TS/A microenvironment, other non-tumoral cell types can play a tumor-promoting role, such as tumor-associated fibroblasts and adipocytes. Through a tumor-stromal cell co-injection model, novel candidate tumor-associated genes were identified in tumor-associated fibroblasts. The most studied gene was tubulin tyrosine ligase: its downregulation in tumor-associated fibroblasts promoted TS/A tumor growth [64]. Co-culture of TS/A cells with adipocytes caused an increased lipid content in TS/A cells and an increased lung colonization ability [65]. The release of free fatty acids from lipid droplets is mediated by an adipose triglyceride lipase-dependent lipolytic pathway, that was proposed as a potential therapeutic target. The metabolic cross-talk between tumor cells and tumor-associated adipocytes could favor epithelial-mesenchymal transition and increase tumor invasiveness.

TS/A cells, like other tumor cell lines, secrete membrane vesicles of endosomal origin called “exosomes”, with contradictory roles in tumor biology. Exosomes could have some immunostimulatory effect, since they carry tumor antigens which can be transferred to dendritic cells and cross-prime cytotoxic T lymphocytes [66]. However, exosomes mainly exerted a potent immunosuppressive anti-tumor immune response through suppression of NK cell function [67] and inhibition of differentiation of bone marrow dendritic cells [68]. Tumor-derived exosomes released from irradiated TS/A cells showed an altered molecular composition and were able to transfer dsDNA to dendritic cells and stimulate upregulation of costimulatory molecules and STING-dependent activation of IFN-I [69].

## 5. Gene Therapy Studies

TS/A cells were easily transduced both with naked DNA and viral systems, generating good and stable transgene expression of secreted factors or membrane molecules. Most gene therapy approaches were performed to directly increase TS/A immunogenicity with the purpose to use engineered cells as anticancer vaccines (Table 2).

Genes for a variety of cytokines, costimulatory molecules and major histocompatibility complex (MHC) antigens were inserted and stably expressed in TS/A cells. Cytokine transduction in TS/A cells was often performed isolating clones with different levels of cytokine production, and this allowed to study the dose-related effects, such as the minimal cytokine release level required to significantly impact on tumor growth and immunogenicity and the potential side effects of highly-releasing cells. As an example, IFN-γ transduction led to isolate clones with cytokine production ranging from a few IU/ml up to a very high expressor clone (releasing 6000 IU/mL), likely the highest transduced expression ever obtained. Such a panel of IFN-γ releasing clones showed a dose-related growth inhibition and immunogenicity, but also showed potentially important side effects, such as increased lung colonizing ability and other systemic effects [12,136].

The wide portfolio of TS/A cells transduced with different cytokine genes allowed to understand the role played by each cytokine in the modulation of tumor infiltrate composition and its impact on tumor growth [137]. A major role for granulocytes in cytokine-induced tumor debulking was unexpectedly found, along with a continuous cross-talk between leukocytes and lymphocytes. The transduced cytokine drove the composition of the reactive cells elicited, the efficacy of the anti-tumor reaction and the immune memory against the non-transduced tumor. The increased memory reaction is the basis for the use of gene-engineered cells as anticancer vaccines. On the whole, data obtained with engineered TS/A vaccines (Table 2) showed that the most effective cytokines were IFN-γ and IL-12.

TS/A transduction with GM-CSF was performed only once [74], with almost no effect on tumor growth or immunogenicity. On the contrary, GM-CSF engineering of another murine model (B16 melanoma) gave good results [138] and prompted clinical studies. B16 melanoma did not produce spontaneously GM-CSF whereas TS/A abundantly secreted CSFs [16]. The spontaneous CSF production in TS/A did not hamper tumor growth but likely contributed to the tumor-promoting environment, showing that similar cytokines could play opposite roles in tumors of different origin.

Transduction of genes coding for activating pro-drug enzymes (suicide genes) was performed with the main aim to obtain more immunogenic cancer cell vaccines. It was reported that replicating cells were more immunogenic than dead cells [74], so prodrug activation by suicide gene products could switch off partially replicating cell vaccines after the start of the immune response. However, prodrug-induced cancer cell death itself was found to increase the specific immune response [125]. Suicide genes were also included in oncolytic viruses, to enhance their safety profile [128].

Gene therapy approaches to obtain increased TS/A cancer cell immunogenicity gave interesting but, at the same time, unsatisfactory results. Most approaches actually showed increased immunogenicity, but when challenged in therapeutic set up, a minority of mice could be cured, and only when therapy started at the very early phases of metastatic growth [106,112]. Similar conclusions could be drawn for the variety of gene therapy trials conducted in the last three decades with the purpose of increasing tumor immunogenicity through cytokine or costimulatory gene transduction. Therefore, results obtained with TS/A as well as with other experimental gene therapy models predicted the low efficacy found in trials. Combined gene therapy approaches showed better therapeutic activity and prompted new combination immune-gene therapy approaches [99,111].

Gene transduction was applied to the TS/A model to study cancer biology and cancer gene therapy (Table 3). Transduction of the wild-type p53 gene (p53wt), aiming to restore a correct p53 signaling, was performed in vitro and in vivo with a Canarypox vector carrying p53wt, leading to downstream p21 expression with a proapoptotic effect that caused tumor growth inhibition [21]. Tumor rejection was associated with the generation of a specific antitumor immune response in a sarcoma model but not in TS/A, thus confirming the low immunogenicity of the TS/A model system.

TS/A cells were transduced with luciferase gene and green fluorescent protein (GFP) variants and used in studies on imaging techniques (Table 3). TS/A cells were used as recipient for genes coding exogenous antigens as a surrogate to study features of the corresponding immune response (Table 3).

Silencing approaches were performed with retro- and lenti-viral vectors and recently with CRISPR-Cas9 technology. Through silencing, TGF-β1 released by TS/A cells was found to play a suppressive role on graft-versus-tumor reaction [18].

In search of new genes potentially involved in metastasis of mammary cancer, along with data from human histopathological samples, some studies used TS/A cells for a mechanistic demonstration through silencing approaches. These studies were sometimes performed in parallel with another popular model of murine mammary cancer (4T1), which is more metastatic than TS/A cells (see Section 6). The overexpression of Fragile X mental retardation protein (FMRP) was concordantly related to lung metastases in both models [46]. On the contrary, some disagreement between TS/A and 4T1 was reported concerning the role of the small GTPase Rab27a [146]. Rab27a was involved in exosome secretion. Its silencing inhibited tumor growth and lung metastases in the 4T1 model, but not in TS/A. It should be noted that the authors described TS/A as a non-metastatic tumor model. Since TS/A is actually able to metastasize to lungs, two explanations are possible for such discrepancy: a) Rab27a is not an on/off determinant of metastatic power, but rather a quantitative modulator; b) 4T1 is a clone while TS/A is a polyclonal and heterogeneous cell line. TS/A extensive subculture can have led to drift phenomena with oligoclonal dominance of less metastatic cells, which are well represented in the cell line of origin.

Genetic inactivation through CRISPR/CAS9 technology of the DNA mismatched repair gene *MutL* homologue 1 (MLH1) in TS/A cells, as well as in other non-mammary murine cancer models, led to increased immunogenicity due to accumulation of neoantigens [147]. MLH1-inactivated cells acquired sensitivity to antibodies against checkpoint inhibitors, which now represent the forefront of cancer immunotherapy.

The expression of murine ErbB2 in TS/A cells was exploited to provide experimental evidence of the oncosuppressor role of FoxP3 in mammary cancers, that downmodulated the expression of the ErbB2 oncogene [44]. TS/A cells was also used as a model to study optimization of parameters of gene electrotransfer [50].

## 6. Comparison with Other Mammary Cancer Models

Modeling mammary cancer in mouse to study tumor–host interactions took advantage of several model systems [3,4,166]. Reordering models according to their intrinsic complexity, we can mention transplantable murine tumors, gene-driven mammary carcinogenic models, human cell lines grown in vitro or in vivo as xenografts and patient-derived xenografts and organoids.

Concerning transplantable murine mammary cancer, the most popular cell line is 4T1, derived from a spontaneous mammary cancer arisen in a BALB/cfC3H female [167,168,169]. 4T1 share several features with TS/A and with human mammary cancers, such as low immunogenicity and tumor–host interactions. In fact, several studies were performed using in parallel 4T1 and TS/A (see for example Supplementary Table S1, column N), which were considered as biological replicates and generally gave concordant results. We can focus here on the main differences between the two models. 4T1 is a thioguanine-resistant clone derived from a heterogeneous mammary cancer cell line [167,168]. TS/A is a cell line with heterogeneity spanning from morphology to metastatic ability and to CSF production (and therefore tumor–host interactions), as proven by the in vitro isolation of clones with markedly different features [14,40]. Populations with different abilities to metastasize were also isolated from TS/A through in vivo selection procedures [41]. Heterogeneity is a hallmark of mammary cancer, which comprises morphology, differentiation and metastatic ability, but cloned populations at least partially lose such heterogeneity. 4T1 is a highly aggressive clone, with the ability to give rise to a high number of lung metastases following subcutaneous, intravenous or orthotopic cell injections (see for example [146]). Moreover, 4T1 can metastasize to other organs (such as liver and bone) [170]. TS/A is a cell line provided with metastatic ability but giving rise to moderate number of lung metastases following local growth, subcutaneous and intravenous injections or orthotopic administration [10,54]. The lower metastatic ability of the TS/A model can allow to study a wider range of metastasis modulators.

In the last three decades the research on mammary tumor development and malignancy took advantage of transgenic models. One of the most studied models of gene-driven mammary carcinogenesis was that based on the rat HER2/neu oncogene under the transcriptional control of the Mouse Mammary Tumor Virus (MMTV) promoter [171,172]. Transgenic models recapitulated all the transitions from the normal mammary gland to mammary cancer, both from morphological and molecular points of view, and led to essential advancement in comprehension of the carcinogenic process and development of new therapeutic approaches. The reproducible carcinogenic process observed in transgenic models was exploited to study the prevention of tumor progression, including approaches based on immune strategies [173]. Transgene expression is somewhat artificial, concerning both the xenogeneic origin of the oncogene (rat HER2/neu) and the expression driven by a viral promoter. From an immunological point of view, the fast carcinogenesis and the altered immunoreactivity of mice being tolerant to transgene can be significant differences from the human pathogenetic development of mammary cancer. However, the main problems of transgenic models are time-consuming procedures and costs. Cell lines from spontaneous mammary cancer such as TS/A therefore are still widely employed in studies on biological features and new therapeutic approaches.

Human models for mammary cancer comprise cell lines and xenografts [4,166]. Human breast cancer is a heterogeneous disease that, thanks to biomarkers, can be subdivided in different subtypes with prognostic significance [174,175] and subjected to appropriate treatments. The main advantage of human models is that they can reproduce the heterogeneity among tumors, giving researchers the possibility to choose the correct subtype depending on the aim of the research, while the main constraint of human cell lines and xenografts is the lack of immune tumor–host interactions. To identify which subtypes can be modeled by the different murine mammary cancers, a comparison among gene expression profiles of a panel of murine mammary cancer cell lines including a TS/A variant (clone E1) and profiles of the different human subtypes was performed [13]. E1 showed a non-basal profile, with prevalent features of luminal A and HER2 subtypes (about 50% and 20%–30% probability, respectively). Therefore, a single murine model can mimic a peculiar human subtype, but obviously is limited to the fixed genetic setting of the cell line and does not reflect diverse spectrum of personalized genetic and/or epigenetic alterations of human breast cancers.

To better depict individual mammary cancers, patient-derived xenografts (PDX) [176] and patient-derived organoids (PDO) were proposed [177,178,179]. Such approaches are more compatible with the need of precision oncology and without concern of species difference. PDX do not allow to study immune interactions (since they are grown in immunodeficient mice), and also present other disadvantages such as the low frequency of tumor take, with a bias toward more aggressive subtypes [180], the cost and the time-consuming procedure. PDO are 3D cultures obtained by dissociated tumor tissue which can be co-cultured with human lymphocytes, thus allowing to investigate tumor microenvironment, anticancer immunotherapy, and other aspects including development of novel therapeutics [181,182].

In conclusion, preclinical models of murine mammary cancer cell lines are still widely used thanks to their possibility to focus on tumor–host interactions comprising the role of stroma, the metastatic process and immune responses. The recent burst of immune-based anti-cancer therapies (see for example checkpoint inhibitors [133,147] and CAR-T [183]) likely will take advantage of murine models comprising mammary cancer cell lines. Other advantages are the low cost and time to obtain results. The possibility to study in parallel several cancer cell lines mimicking different breast cancer subtypes could remain a first-line means to study innovative molecular and therapeutic approaches, which will be then tested in individually precise, more complex human models.

## 7. Conclusions

The analysis of the main studies exploiting TS/A as a pre-clinical model of mammary cancer allows to draft a profile spanning from molecular alterations to malignant phenotype and immune interactions. This profile should be considered when designing experiments based on TS/A model. Knowledge of this profile can allow inference about the complexity of human breast cancer.

## Figures and Tables

**Table 1:** TS/A model: main features.

Topics	Cell Variants	Features	Refs
Cytoskeletal markers	E1	CK8-positive	[13]
Cytokine production	TS/A, clones and variants	CSF	[14,15,16,17]
	TS/A	TGF-β1 production (about 4 ng/mL)	[18]
Cytokine receptors	TS/A	IFN-γ receptor (1000/cell)	[19]
Gene alterations	TS/A	Karyotype	[20]
	TS/A and E1	p53 mutated (codon 270 Arg to His)	[13,21]
Gene expression	TS/A	TERT (11,000 RNA copies)	[22]
Hormone sensitivity	TS/A and E1	Estrogen receptor positive	[10,13]
Immunity	TS/A	Low immunogenicity	[10,23,24]
	TS/A and engineered variants	Tumor associated antigen gp70env	[25]
	TS/A	Suppressor activity	[26,27]
	TS/A	Myeloid-derived suppressor cells (MDSC)	[28,29,30,31,32,33,34,35]
	TS/A	NK resistance	[36]
	TS/A	mD52 antigen	[37]
Membrane molecules	TS/A	Core 1 O-glycans	[38]
	TS/A	Muc-1	[39]
	TS/A	Tag72	[39]
Phenotype	TS/A, clones and variants	Heterogeneity (morphology, metastasis)	[10,40,41]
Stem cell markers	TS/A	Sca-1 (Ly6A/E)	[42,43]
Tyrosine Kinase membrane receptors	TS/A	p185erbB2	[39,44]
Others	TS/A	Endoglin-negative	[45]
	TS/A	Fragile X mental retardation protein (FMRP), low expression	[46]
	TS/A	High Mobility Group Box1 (HMGB1)-positive	[47]
	TS/A	Lats2	[48]
	TS/A	ST6Gal activity (present, low)	[49]
	TS/A	TLR9-negative	[50]

**Table 2:** TS/A in gene therapy studies aiming to increase tumor immunogenicity.

Immune Categories	Transgenes	Vector/Transfer Methods	Refs
Chemokines	h-CCL16/LEC	Naked DNA + lipofection	[70,71]
		Adenoviral, in vivo	[59,72]
Cytokines	m-IL2	Naked DNA + electroporation	[11,23,73,74,75,76,77]
	m-IL4	Retroviral	[73,74,78,79,80,81,82,83,84,85]
	m-IL5	Retroviral	[86,87]
	m-IL6	Retroviral	[74,80,87]
	m-IL7	Naked DNA + electroporation	[73,74,80,81,88,89]
	m-IL7	Adenoviral	[90]
	m-IL10	Naked DNA + electroporation	[74,91]
	m-IL12 p35 and p40	Naked DNA, in vivo	[92,93]
		Naked DNA + gene gun	[94]
		Naked DNA + lipofection	[95]
		Retroviral	[96,97]
		Canarypox	[98]
		Naked DNA + gene gun, in vivo	[24]
	h-IL13	Naked DNA + calcium phosphate	[99]
	m-IL15	Adenoviral	[100]
	IL15	Naked DNA + lipofection	[95,101]
	Pro-IL18 and ICE	Naked DNA + gene gun	[94]
	m-IL21	Naked DNA + lipofection	[102]
	m-IFNα1	Naked DNA + electroporation	[80,82,103,104,105,106]
	m-IFNα1	Naked DNA + gene gun	[107]
	m-IFNα4	Naked DNA + polymer	[108]
	m-IFNβ	Naked DNA + calcium phosphate	[109]
	m-IFNγ	Naked DNA + lipofection	[12,74,80,99,110,111,112,113]
	m-GMCSF	Retroviral	[74]
	m-TNFα	Retroviral	[74,80]
Membrane molecules	B7-1/CD80	Naked DNA	[114]
		Naked DNA + electroporation	[115,116]
		Retroviral	[81,89]
	B7-2/CD86	Naked DNA + electroporation	[115,116]
	Allogeneic MHC	Naked DNA + calcium phosphate	[111,117]
	CD70 (CD27L)	Retroviral	[118,119]
	CD153 (CD30L)	Retroviral	[118]
	CD154 (CD40L)	Retroviral	[118,120]
	TRAIL/APO2L	Naked DNA + lipofection	[121]
	LAG-3 and LAG5	Naked DNA + electroporation	[122,123]
	CCR7	Naked DNA + calcium phosphate	[124]
Suicide genes	Cytosine deaminase	Retroviral	[112,125]
		Naked DNA	[126]
		VSV (oncolytic)	[127,128]
	HSV-Thymidine kinase	Naked DNA	[104]
		Retroviral	[129]
		Naked DNA + lipofection	[130]
Others	CIITA	Naked DNA + lipofection	[131,132,133]
	GBP1	Retroviral (conditional) + naked DNA	[134]
	m-IRF1	Adenoviral	[135]

**Table 3:** TS/A in transduction studies of cancer biology.

Gene Categories	Transgenes	Vector/Transfer Methods	Refs
Oncosuppressors	m-p53wt	Canarypox	[21]
	m-p53wt/mut	VSV (oncolytic)	[139]
Reporter genes	Luciferase	Naked DNA	[140,141]
	β-galactosidase	Naked DNA + polyfection	[142]
	GFP	Adenoviral	[143]
	GFP	Lentiviral	[46]
	EGFP	Lentiviral	[54]
	EGFP	Naked DNA + electroporation	[50]
	EGFP (driven by p21 or CMV promoter)	Naked DNA + electroporation	[144,145]
Silencing	antisense m-TGF-β1	Retroviral	[18]
	Rab27a	Lentiviral	[146]
	Mlh1	CRISPR-Cas9	[147]
	FoxP3	Lentiviral siRNA	[44]
	fragile X mental retardation protein (FMRP)	Lentiviral shRNA	[46]
Surrogate antigens	β-galactosidase	Retroviral	[81,148,149,150]
	Hemagglutinin	Naked DNA + lipofection	[151,152]
	Leishmania receptor for activated C kinase (LACK)	Naked DNA	[153,154,155]
	Mycobacterial cell wall-associated 19-kDa lipoprotein		[156]
	Ovalbumin	Naked DNA	[157,158]
Others	Chromogranin A (Vasostatin-1 fragment)	Naked DNA + electroporation	[159,160,161]
	Extracellular domain of receptor tyrosine kinase Tie2/TEK (ex-TEK)	Naked DNA + calcium phosphate precipitation	[162]
	Apelin	Naked DNA + polyfection	[163]
	Interferon-regulatory factor-1 (IRF-1)	Adenoviral	[43]
	α1,2fucosyltransferase	Naked DNA + lipofection	[164]
	P27VP22	Naked DNA + polyfection	[165]

## Supplementary Materials

The following are available online at https://www.mdpi.com/2072-6694/11/12/1889/s1, Table S1: Comprehensive list of papers using TS/A cell variants.
